# Supplementary material for: A Scoping Review on How to Make Hospitals Health Literate Healthcare Organizations
Source: Int J Environ Res Public Health. 2020 Feb 6;17(3):1036. doi: 10.3390/ijerph17031036 (PMC7037285; doi:10.3390/ijerph17031036)
Supplement: Supplementary file 1 [file ijerph-17-01036-s001.pdf]

Table S1: Primary Studies

Autor, year	Country	Study design	Aim	Attributes	Conclusions	Title	Outcomes	Intervention	Intervention code	Outcome code	Ref
Bailey, Stacy Cooper 2012	Usa	RCT	To evaluate the efficacy of health literacy informed, multilingual prescrition (Rx) instructions (the ConcordantRx instructions) to improve Rx understanding, regimen dosing and regimen consolidation in comparison to standard, language-concordant Rx instructions	8,9	Subjects receiving the ConcordantRx instructions demonstrated significantly greater Rx understanding, regimen dosing and regimen consolidation in comparison to those receiving standard instructions	Evaluation of language concordant, patient-centered drug label instructions	-understanding prescriptions -regimen dosing -regimen consolidation	multilingual prescrition (Rx) instructions	1a	1a	18
Bell, Susan 2016	USA	RCT	To determine the effect of a tailored, pharmacist-delivered, health literacy intervention on unplanned health care utilization, including hospital readmission or emergency room (ER) visit, following discharge.	6,7,8,9	Tailored, pharmacist-delivered health literacy-sensitive intervention did not reduce postdischarge unplanned health care utilization overall. The intervention was effective among patients with inadequate health literacy, suggesting that targeted practice of pharmacist intervention in this population may be advantageous	Effect of Pharmacist Counseling Intervention on Health Care Utilization Following Hospital Discharge: A Randomized Control Trial	. time to first unplanned health care event, defined as hospital readmission or an ER visit within 30 days of discharge	. Multifaceted: pharmacist-assisted medication reconciliation, inpatient pharmacist counseling, low-literacy adherence aids, and individualized telephone follow-up after discharge	1ab	1c	19
Bickmore, T. W. 2009	USA	quasi-experimental	To describe the development methodology, design rationale, and two iterations of user testing of an empathic virtual nurse interface for educating and counseling hospital patients with inadequate health literacy	7,8,9	Hospital patients with low health literacy found the system easy to use, reported high levels of satisfaction, and most said they preferred receiving the discharge information from the agent over their doctor or nurse. Patients also expressed appreciation for the time and attention provided by the virtual nurse, and felt that it provided an additional authoritative source for their medical information.	Taking the Time to Care: Empowering Low Health Literacy Hospital Patients with Virtual Nurse Agents	. perception of satisfaction and ease of use (patient)	. virtual nurse interface	1a	1b	20
Cawthon, Courtney 2012	USA	quasi-experimental	To test the intervention arm of the Pharmacist Intervention for Low Literacy in Cardiovascular Disease (PILL-CVD) study who completed a telephone survey about the helpfulness of different components of the PILL-CVD intervention, which included medication reconciliation, inpatient	7,9	Patients with limited health literacy indicated the greatest benefit. Patients also reported feeling more comfortable speaking with their outpatient providers about their medications after receiving the intervention.	Improving care transitions: the patient perspective	.perception of helpfulness (for patient) .perception of comfortablenes (for patients)	. medication reconciliation, inpatient counseling, simple adherence aids, and telephone follow-up	1ab	1b	21

			counseling, simple adherence aids, and telephone follow-up.								
Choi, J. 2010	USA	Validation/usability	To address low literacy of Neonatal Intensive Care Unit parents, thanks to multimedia educational Website using visual aids (e.g., pictographs, photographs), voice-recorded text message in addition to a simplified text was developed	8	The findings suggest that using concrete and realistic pictures and pictographs with clear captions would maximize the benefit of visuals	Web-based education for low-literate parents in Neonatal Intensive Care Unit: development of a website and heuristic evaluation and usability testing	.End-users' performance .perception of ease of use and usefulness (patient)	. multimedia educational Website using visual aids (e.g., pictographs, photographs), voice-recorded text message in addition to a simplified text	1a	1ab	22
Cordasco, K. M. 2009	USA	RCT	To develope and evaluate a low-literacy medication education tool to improve medication adherence in cardiac patients	8,9	The evaluation did not demonstrate the tool to have any effect on self-reported medication adherence, patients who received the schedule self-reported their medication adherence more accurately, perhaps indicating improved understanding of their medication regimen and awareness of non-adherence	A Low-Literacy Medication Education Tool for Safety-Net Hospital Patients	.medication adherence and knowledge .acceptability for nurses	. medication education tool, customizable for each patient, featuring instruction-specific icons and pictures of pills.	1a	1a 2b	23
Dankner, R. 2015	Israel	quasi-experimental	Patients in the intervention arm received written and oral explanations on cardiac rehabilitation benefits and eligibility, and a follow-up telephone call 2 weeks after hospital discharge	7,8	Participation in cardiac rehabilitation almost doubled following a low cost intervention with significant effects on subpopulations that have been underrepresented in cardiac rehabilitation programs	A controlled intervention to increase participation in cardiac rehabilitation	.Adherence to rehabilitation	. Multifaceted: written and oral explanations on cardiac rehabilitation benefits and eligibility, and a follow-up telephone call 2 weeks after hospital discharge	1ab	1a	24
Foertsch, L. Y. 2016	USA	quasi-experimental	To develop, implement, and evaluate a teaching tool for self-assessment of surgical incisions after laparotomy surgery	8,9	The revised teaching program (text and pictures) and use of a handheld mirror improved patient confidence in self-assessing an incision and increased ease in detecting an SSI.	Evaluation of a Surgical Site Discharge Teaching Tool Using Pictures and a Mirror	.comprehension of instructions .felt that instructions were clearly stated . were confident in their ability to identify normal healing versus a surgical site infection (SSI)	. teaching program (text and pictures)	1a	1a	25

							and aboutnotifying physicians				
Gallagher, R. 2013	Australia	quasi-experimental	To examine an individual educational intervention on knowledge of heart attack warning signs and specific chest pain action plans for people with coronary heart disease.	8,9	A brief education session using a single standardised tool and adapted to a patient assessment is effective in improving knowledge of potential AMI symptoms and appropriate responses in cardiac rehabilitation up to two months following.	A pre-test post-test study of a brief educational intervention demonstrates improved knowledge of potential acute myocardial infarction symptoms and appropriate responses in cardiac rehabilitation patients	.knowledge of potential acute myocardial infarction symptoms and appropriate responses in cardiac rehabilitation patients	. a single standardised easy to understand tool	1a	1a	26
Herman, A. 2009	USA	quasi-experimental	To measure the impact of a simple parent health literacy intervention on emergency department and primarycare clinic usage patterns	8, 9	Before and after comparisons demonstrated a 13% reduction in the percentage of respondents who stated they would go to the emergency department first if their child became sick	Impact of a health literacy intervention on pediatric emergency department use	.knowledge of how to use emergency department .reported visits in emergency department in the last 6 months .proportion of parents who would take their child to the emergency department for low-acuity scenarios	. participants were educated about how to use the health aid book What to Do When Your Child Gets Sick (easy to understand tool)	1a	1ac	27
Hopmans, W. 2014	The Netherlands	Descriptive	To applie a qualitative user testing during the development of a patient information website on stereotactic ablative radiotherapy	4,7,8	Findings indicate that input from patients and their relatives allows for a more comprehensive and usable website for providing treatment information	Communicating cancer treatment information using the Web: utilizing the patient's perspective in website development	. Patient preference (qualitative analysis)	. A qualitativeuser test of the website was performed in 18 subjects, followed by an additional evaluation by users after website redesign (N = 9)	1a	1b	38

Hu, H. 2013	Taiwan	quasi-experimental	The effectiveness of three education programs (Group 1 subjects read the package insert; Group 2 read a photograph-designed educational sheet; and Group 3 received a face-to-face medication education from a pharmacist with the photograph-designed educational sheet)for pediatric patients' caregivers was evaluated using a questionnaire comprised of 12 questions.	8,9	The study demonstrated that when compared to reading a package insert or education sheet, a pharmacist's verbal education with photographic education materials was significantly more effective and time-saving in providing caregivers with the correct knowledge of oral antibiotic suspensions in pediatrics	Effectiveness of Education Programs About Oral Antibiotic Suspensions in Pediatric Outpatient Services	.knowledge about oral antibiotic (caregivers)	. three education programs (Group 1 subjects read the package insert; Group 2 read a photograph-designed educational sheet; and Group 3 received a face-to-face medication education from a pharmacist with the photograph-designed educational sheet)	1ab	1a	29
Huang, C. H. 2016	USA	quasi-experimental	To examine the feasibility of a multicomponent advance care planning (ACP) intervention program that integrates motivational interviewing, evidence-based ACP facilitation program (Respecting Choices), and health-literacy adjusted advance directives (AD).	7,8	All participants (n = 30) reported high satisfaction (M= 4.81, SD = 0.44, max score = 5) and increased intent to complete an advance directives (AD) at postintervention. A significant increase in knowledge on AD from baseline to postintervention was observed in the intervention group	A Pilot Feasibility Intervention to Increase Advance Care Planning among African Americans in the Deep South	.high satisfaction<br>. increased intent to complete an advance directives<br>. Increased knowledge on AD from baseline to postintervention	. multicomponent advance care planning (ACP) intervention program that integrates motivational interviewing, evidence-based ACP facilitation program (Respecting Choices), and health-literacy adjusted advance directives (AD)	1ab	1ab	30
Jibaja-Weiss, M. L. 2011	USA	RCT	To compare a computerized, multimedia, interactive patient decision aid (CPtDA) utilizing edutainment (a combination of soap opera episodes and interactive learning modules [ILMs]) along with usual care to receiving usual care alone for early stage breast cancer surgery in low health literacy patients	7,8	Entertainment education may be a desirable strategy for informing lower health literate women about breast cancer surgery options.	Entertainment education for breast cancer surgery decisions: a randomized trial among patients with low health literacy	.more patient choose mastectomy rather than breast-conserving surgery<br>. Satisfaction with the surgical decision or the decision-making process<br>.better informed and<br>clearer about surgical options	. a computerized, multimedia, interactive patient decision aid (CPtDA) utilizing edutainment (a combination of soap opera episodes and interactive learning modules [ILMs])	1a	1ab	31

Joseph, G. 2010	USA	Descriptive	To qualitatively evaluate participants' experience with and perceptions of a genetic education program as an adjunct to genetic counseling	7,8,9	In a post-session counselor questionnaire, counselors' rating of the patient's preparedness before the session was significantly higher for patients who viewed CREdIT prior to their appointments than for other patients. This novel educational tool fills a gap in HBOC education by tailoring information to women of lower literacy and diverse ethnic/racial backgrounds	Pre-counseling Education for Low Literacy Women at Risk of Hereditary Breast and Ovarian Cancer (HBOC): Patient Experiences Using the Cancer Risk Education Intervention Tool (CREdIT)	.patients preaparadness	. a computer-based (non-interactive) slide presentation	1a	1a	32
Kheir, N. 2014	Qatar	Descriptive	To develop pictograms illustrating selected medicine label instructions and to evaluate comprehension of the pictograms or conventional text supported with verbal instructions in foreign workers with low literacy skills.	8	Medication labels consisting of simple pictorials supported by verbal instructions were better comprehended by individuals with low literacy skills than labels with written plus verbal instructions	Development and evaluation of pictograms on medication labels for patients with limited literacy skills in a culturally diverse multiethnic population	. Label comprehension	. pictograms illustrating selected medicine label instructions	1a	1a	33
Kripalani, S. 2012	USA	RCT	The Pharmacist Intervention for Low Literacy in Cardiovascular Disease (PILLCVD) study sought to determine the effect of a tailored intervention on the occurrence of clinically important medication errors after hospital discharge. The intervention consisted of 4 components – pharmacist-assisted medication reconciliation, tailored inpatient counseling by a pharmacist, provision of low-literacy adherence aids, and individualized telephone follow-up after discharge	7, 9	Clinically important medication errors were present among half of patients after hospital discharge and were not significantly reduced by a health-literacy sensitive, pharmacist delivered intervention.	Effect of a pharmacist intervention on clinically important medication errors after hospital discharge: a randomized trial	.number of clinically important medication errors per patient during the first 30 days after hospital discharge .preventable or ameliorable adverse drug events, as well as potential adverse drug events	. multifaceted: pharmacist-assisted medication reconciliation, tailored inpatient counseling by a pharmacist, provision of low-literacy adherence aids, and individualized telephone follow-up after discharge	1ab	1c	34

Kripalani, Sunil 2007	USA	RCT	to describe the development, implementation, and preliminary evaluation of an illustrated medication schedule (a ‘‘pill card’’) that depicts a patient’s daily medication regimen using pill images and icons	8,9	Nearly all patients considered an illustrated medication schedule to be a useful and easily understood tool to assist with medication management. Patients with limited literacy skills, educational attainment, or cognitive function referred to the aid with greater frequency	Development of an illustrated medication schedule as a low-literacy patient education tool	. .perception of ease of use and usefulness (patient)<br>. Frequency of use of the aid	. illustrated medication schedule (a ‘‘pill card’’)	1a	1a	35
Kuppermann, M. ; 2014	USA	RCT	To analyze the effect of a decision support guide and elimination of financial barriers to testing on use of prenatal genetic testing and decision-making among women of varying literacy and numeracy levels A computerized, interactive decision support guide and access to prenatal testing with no out-of-pocket expense (n=357) or usual care as per current guidelines (n=353)	6, 7, 8	Women who were randomized to the intervention group would undergo invasive diagnostic testing at a lower rate than women randomized to the control group, due to better understanding	Effect of enhanced information, values clarification, and removal of financial barriers on use of prenatal genetic testing: a randomized clinical trial	.better understanding<br>. Rate of patient who undergo invasive diagnostic testing	. A computerized, interactive decision support guide and access to prenatal testing with no out-of-pocket expense	1a	1a	36
Marcantoni, Jodie R. 2014	USA	quasi-experimental	Preintervention patients received usual discharge teaching. Postintervention patients received an educational brochure written at a seventh-grade reading level.	8, 9	There was a significant improvement in follow-up adherence in the postintervention group compared to the preintervention group (P = .02). Readmissions were similar in each group (P = .17). Treatment failure was the most common reason for readmission in both groups	Using health literacy guidelines to improve discharge education and the post-hospital transition: a quality improvement project	.follow-up adherence<br>.n. of readmissions	. educational brochure written at a seventh-grade reading level	1a	1ac	37
Miller, M. J. 2011	USA	Descriptive	This research sought to describe and compare perceptions of consent-related health communication between surgical patients undergoing procedures at facilities that did and did not adopt a new health literacy based consent form and process.	4, 5, 6, 7, 8, 9	The consent process can be refined to stimulate communication and comfort with asking questions, and promote use of health literacy based techniques (i.e., teach-back) in the perioperative care setting. Adopting a health literacy based informed consent process promotes patient safety and supports health providers’ obligations to communicate in simple, clear, and plain language.	Improving Patient-Provider Communication for Patients Having Surgery: Patient Perceptions of a Revised Health Literacy-Based Consent Process	.comfortable asking questions about their surgery<br>.nurses asked them to restate the type of surgery being performed in their own words	.a new health literacy based consent form and process	1ab	1a 2a	38

Mueller, S. K. 2015	USA	quasi-experimental	To examine the impact of the use of electronic, patient-friendly, templated discharge instructions on the readability of discharge instructions provided to patients at discharge.	8, 9	The use of electronically available templated discharge instructions was associated with better readability than the use of clinician-generated discharge instructions. Use of electronically available templated discharge instructions may be a viable option to improve the readability of written material provided to patients at discharge, although the library of available templates requires expansion	Readability of patient discharge instructions with and without the use of electronically available disease-specific templates	.better readability	.electronic, patient-friendly, templated discharge instructions	1a	3c	39
Murray, M. D. 2007	USA	RCT	To determine whether a pharmacist intervention improves medication adherence and health outcomes compared with usual care for low-income patients with heart failure	7, 8, 9	A pharmacist intervention for outpatients with heart failure can improve adherence to cardiovascular medications and decrease health care use and costs, but the benefit probably requires constant intervention because the effect dissipates when the intervention ceases.	Pharmacist intervention to improve medication adherence in heart failure - A randomized trial	.adherence to cardiovascular medications<br>. Health care use and cost	. educational intervention by a pharmacist. The pharmacist provided patient-centered verbal instructions and written materials about the medications	1ab	1a 3d	40
Press, V. G. 2012	USA	RCT	To compare two strategies for teaching inhaler use to hospitalized patients with asthma or chronic obstructive pulmonary disease (COPD).	6,8,9	TTG appears to be more effective compared with BI.	Teaching the use of respiratory inhalers to hospitalized patients with asthma or COPD: A randomized trial	. metereddose inhaler (MDI) misuse post-intervention<br>.Diskus® misuse<br>. self-reported inhaler technique confidence<br>. prevalence of 30-day health-related events	. Participants were randomized to brief intervention [BI]: single-set of verbal and written step-by-step instructions, or, teach-to-goal [TTG]: BI plus repeated demonstrations of inhaler use and participant comprehension assessments (teach-back).	1ab	1ac	41
Roberts, N. J. 2011	England	quasi-experimental	This study was designed to produce a comprehensible pictorial COPD action plan for use by patients and health care professionals.	4,8	Demonstrate that pictograms designed specifically for a COPD action plan are understood by a random cross-section of COPD patients	Evaluation of a paper and electronic pictorial COPD action plan	.Guessability and translucency scores<br>. Patients knowledge	. To produce a comprehensible pictorial COPD action plan	1a	1a	42
Sahm, L. J. 2012	Ireland	RCT	To assess the efficacy of patient-centered label (PCL) instructions on the knowledge and comprehension of prescription drug use compared to standard instructions.	8,9	PCL instructions were more likely to be correctly interpreted than the standard instructions	What's in a label? An exploratory study of patient-centered drug instructions	.correct interpretation	. patient-centered label (PCL) instructions	1a	1a	43

Send, A. F. J. 2014	Germany	quasi-experimental	This study assessed the effect of providing an enhanced medication plan (EMP) to patients during patientphysician conversation at hospital discharge and evaluated it' s immediate impact on patient knowledge on pharmacotherapy.	8,9	The provision of an EMP improves information transfer and therefore increases the patients' knowledge of their individual drug treatment without prolonging the overall discharge process.	Pilot study to assess the influence of an enhanced medication plan on patient knowledge at hospital discharge	.knowledge patient	. enhanced medication plan. The layout of the EMP was designed to allow the electronically supported integration	1a	1a	44
Seth, Natasha 2016	USA	quasi-experimental	To implement written educational materials at an appropriated literacy level to communicate key information to the underserved gynecological surgery patients	8,9	Decreased readmissions, empowerment of patients, and cost effective care.	A health literacy strategy to empower the underserved gynecological surgery patients to reduce 7-day readmissions	.deacrease of readmissions	. written educational materials at an appropriated literacy level	1a	1c	45
Sox, C. M. 2010	USA	quasi-experimental	To create an interface for parents of children with attention-deficit hyperactivity/disorder (ADHD) to enter disease-specific information to facilitate data entry with minimal task burden	7	Our patient-centered design process produced a usable ADHD-specific personal health application that minimizes the burden of data entry	Patient-centered design of an information management module for a personally controlled health record	. Mean time on task	. ADHD-specific personal health application to support data entry into a personally controlled health record (PCHR)	1a	1a	46
Sudore, Rebecca L. 2007	USA	RCT	To determine whether an advance directive redesigned to meet most adults' literacy needs (5th grade reading level with graphics) was more useful for advance care planning than a standard form (>12th grade level)	8	The redesigned advance directive was rated more acceptable and useful for advance care planning and was preferred over a standard form. It also resulted in higher six month completion rates.	An advance directive redesigned to meet the literacy level of most adults: A randomized trial	.acceptability<br>. Completion rates	.advance directive redesigned	1a	1ab	47
van der Vaart, R. 2014	The Netherlands	quasi-experimental	To measure the use, satisfaction and impact of a web portal which provides patients with rheumatoid arthritis home access to their electronic medical records (EMR).	7	The current portal succeeded in offering patients access to their EMR in a usable and understandable way. While its true impact is difficult to grasp, a relevant portion of the patients felt more involved in their treatment due to the web portal	Impact of patient-accessible electronic medical records in rheumatology: use, satisfaction and effects on empowerment among patients	. Satisfaction with patient portal<br>. Subjective impact of the patient web portal	. a web portal	1a	1b	48
Williams, Allison 2012	Australia	RCT	A translated, multifactorial intervention, consisting of a medication review, a short PowerPoint presentation and motivational interviewing designed to improve medication self-efficacy and adherence, was tested in a randomised controlled	6,8,9	There were no significant differences in medication self-efficacy or adherence between the intervention and control groups at three, six and 12 months post-baseline.	Working with CALD groups: testing the feasibility of an intervention to improve medication self- management in people with kidney disease, diabetes, and cardiovascular disease	. medication self-efficacy<br>. Adherence	. Multifaceted: medication review, a short PowerPoint presentation and motivational interviewing	1ab	1a	49

			trial (RCT) with 12 months follow-up post-baseline.							
Wolf, M. S. 2014	USA	quasi-experimental	To evaluate the effectiveness of patient-centered strategies for the design of Med Guides to improve comprehension	4,8	The application of evidence-based practices to the redesign of Med Guides significantly improved patient comprehension.	Comparative Effectiveness of Patient-centered Strategies to Improve FDA Medication Guides	. Comprehension	. patient-centered strategies for the design of Med Guides	1a2b3b	1a	50
Yin, H. S. 2014	USA	quasi-experimental	To examine the degree to which recommended provider counseling strategies, including advanced communication techniques and dosing instrument provision, are associated with reductions in parent liquid medication dosing errors	6,8	Advanced counseling and instrument provision in the Emergency Department were associated with decreased errors	Liquid medication dosing errors in children: Role of provider counseling strategies	. Dosing errors	multifaceted: advanced communication techniques and dosing instrument provision	1ab	1a	51
Yin, H. S. 2008	USA	RCT	To evaluate the efficacy of a pictogrambased health literacy intervention to decrease liquid medication administration errors by caregivers of young children.	8,9	Plain language, pictogram-based intervention used as part of medication counseling resulted in decreased medication dosing errors and improved adherence among multiethnic, low socioeconomic status caregivers	Randomized controlled trial of a pictogram-based intervention to reduce liquid medication dosing errors and improve adherence among caregivers of young children	. Dosing errors<br>. Adherence	. Pictogram based health literacy intervention	1a	1a	52
Zullig, L. L. 2014	USA	Validation/usability	To determine whether antihypertensive medication adherence could improve using a Meducation1 technology health literacy intervention(calendars, containing: (1) medication name; (2) time of day, including a pictorial display, that medication should be consumed; (3) number of times daily that medication should be taken; (4) dose of medication administered; and (5) clinical indication for medication	8	A health literacy intervention may be a feasible mechanism to improve cardiovascular-related medication adherence and outcomes	A health literacy pilot intervention to improve medication adherence using Meducation(R) technology	. self-reported medication adherence	.Meducation1 technology health literacy intervention(calendars, containing: (1) medication name; (2) time of day, including a pictorial display, that medication should be consumed; (3) number of times daily that medication should be taken; (4) dose of medication administered; and (5) clinical indication for medication)	1a	1a	53

Axelrod, D. A. 2017	USA	Validation/usability	A mobile iOS based application (app) including animated patient education and individualized risk adjusted outcomes following kidney transplants with varying donor characteristics and DSA waiting times was piloted in 2 large US transplant programs with a diverse group of renal transplant candidates	7,8,9	This pilot project confirmed the benefit and cultural acceptability of this educational tool, and further refinement will explore how to better communicate the risks and benefits of non-standard donors.	Cultural competency of a mobile, customized patient education tool for improving potential kidney transplant recipients' knowledge and decision-making	. Knowledge<br>. Perception of appropriateness for their race/ethnicity<br>. Acceptability	. mobile iOS based application (app) including animated patient education and individualized risk adjusted outcomes following kidney transplant	1a	1ab	54
Davis, Terry C. 2017	USA	quasi-experimental	To improve patient colonoscopy bowel preparation with a newly developed simplified instruction sheet in a safety-net hospital system	8	A no-cost simplified colonoscopy instruction sheet improved bowel preparation among patients in an academicsafety-net health system	Impact of Health Literacy-directed Colonoscopy Bowel Preparation Instruction Sheet	. Adequacy of preparation	. instruction sheet	1a	1a	55
Felicitas-Perkins, J. Q. 2017	USA	quasi-experimental	to determine the effect of a culturally-tailored educational DVD on cancer clinical trial participation among Filipino cancer patients	4,7,8	A multilingual educational DVD to supplement clinical trial education may positively influence Filipino cancer patients to move forward with the decision to join a cancer clinical trial	A Pilot Study to Determine the Effect of an Educational DVD in Philippine Languages on Cancer Clinical Trial Participation among Filipinos in Hawai'i	. number of participants enrolled into a clinical trial	. educational DVD	1a	1b	56
Lindquist, L. A. 2017	USA	RCT	Investigate the effect of PlanYourLifespan.org (PYL) on knowledge of posthospital discharge options	7,8	Seniors who used PYL demonstrated an increased understanding of posthospitalization and home services compared to the control group	Helping seniors plan for posthospital discharge needs before a hospitalization occurs: Results from the randomized control trial of planyourlifespan.org	. Knowledge	. PlanYourLifespan.org (PYL) a national, publicly available tool that provides education on posthospital therapy choices and local home-based resources	1a	1a	57

McWilliams, A. 2018	USA	case study	To describe a case study of a pediatric asthma shared decision-making health IT solution's development and demonstrate a methodology for engaging actual patients and families in IT development. Perspectives are shared from the vantage point of the research team and a parent of a child with asthma, who participated on the development team.	4,7,8	Using this methodology, we successful partnered with asthma patients and families to create an interactive, digital solution called Carolinas Asthma Coach. Carolinas Asthma Coach incorporates SDM principles to elicit patient information, including goals and preferences, and provides health-literate, tailored education with specific guideline-based recommendations for patients and their providers. Of the patients, caregivers, and providers surveyed, 100% (n=60) said they would recommend Carolinas Asthma Coach to a friend or colleague. Qualitative feedback from users provided support for the usability and engaging nature of the app.	Patient and Family Engagement in the Design of a Mobile Health Solution for Pediatric Asthma: Development and Feasibility Study	. Feasibility, usability (Qualitative feedback)	. user-centric design principles: (1) a Development Workgroup consisting of patients, caregivers, and providers who met regularly with the research team; and (2) “real-world users” consisting of patients, caregivers, and providers	1a	3c	58
Phillippi, J. C. 2018	USA	RCT	To evaluate the feasibility and utility of e-consent in the first year of a multiyear clinical trial involving pregnant women	4,7,9	E-consent is feasible and easy to use with pregnant women and may expedite enrollment of a representative sample.	Electronic Informed Consent to Facilitate Recruitment of Pregnant Women Into Research	. Comparison of racial/ethnic distribution of paients	. e-consent	1a	1ab	59
Press, V. G. 2017	USA	quasi-experimental	To develop and pilot a virtual Teach-To-Goal (V-TTG) inhaler skill training module, using innovative adaptive learning technology	7,8	This study is the first to demonstrate the efficacy of adaptive V-TTG learning to teach inhaler technique	Virtual Teach-To-Goal™ Adaptive Learning of Inhaler Technique for Inpatients with Asthma or COPD	. proportion of participants with inhaler misuse post versus pre V-TTG<br>. mastery, self-efficacy<br>. perceived versus actual inhaler skills	. a virtual Teach-To-Goal (V-TTG) inhaler skill training module	1a	1a	60

Priegue, M. 2017	Spain	quasi-experimental	Pharmacist intervention, based on two pharmacist-visit programs and development of information for patients, was applied to patients under pharmacological treatment in a non-endemic country	6,8,9	It was not possible to demonstrate that pharmacist intervention that includes in-depth patient information about possible ADRs and their management could increase adherence and treatment completion rates. However, pharmacist interventionhelped to better detect and control ADRs that could lead to generating better health outcomes in Chagas disease	Pharmacist intervention in patients receiving treatment for Chagas disease: An emerging challenge for non-endemic countries	. % of patients who completed the treatment<br>. detection of ADRs	. Multifaceted: Information sheet and Two-visit hospital pharmacist regimen: Visit 1 (day 1): Structured pharmacist-patient hospital interview on day 1 for delivering oral information, written information, and medication for 1 month. Visit 2 (day 30): Pharmacist-patient hospital interview to review the oral information given on day 1, ask about compliance and ADRs, and provide medication for 1 month pharmacist-visit program	1ab	1a	61
Spellecy, R. 2018	USA	RCT	To evaluate a 2-column easy-to-read informed consent (ETRIC) form that incorporates elements of health literacy and readability in participants and centers participating in Blood and Marrow Transplant Clinical Trials Network (BMT CTN) clinical trials	8,9	There was no significant association between the consent type and QuIC-A score	Easy-to-Read Informed Consent Form for Hematopoietic Cell Transplantation Clinical Trials: Results from the Blood and Marrow Transplant Clinical Trials Network 1205 Study	. comprehension score<br>. dominant themes identified on qualitative analyses	. 2-column easy-to-read informed consent (ETRIC)	1a	3a	62
Srisuk, Nittaya 2017	Thailand	RCT	To evaluate a heart failure education programme developed for patients and carers	6,7,8,9	Addressing a significant service gap in rural Thailand, this familybased heart failure programme improved patient knowledge, self-care behaviours and health-related quality of life and carer knowledge and perceived control	Randomized controlled trial of family-based education for patients with heart failure and their carers	. knowledge scores<br>self-care maintenance<br>. Confidence scores<br>. health-related quality of life scores<br>. self-care management scores<br>. perceived control scores	. heart failure education programme:6-month programme comprising one face-to-face education, counselling session, provision of a heart failure manual and DVD and telephone follow-up. The education session was conducted in the hospital outpatient clinic teaching room and lasted 40–60 minutes.	1ab	1abc	63

Unaka, N. 2017	USA	Case study	To increase the percentage of discharge instructions written at 7th grade level or lower for hospital medicine patients from 13% to 80% in 6 months	8,9	Through sequential interventions over 6 months, the percentage of discharge instructions written at 7th grade or lower readability level increased from 13% to 98% and has been sustained for 4 months. The reliable use of the EHR templates was associated with our largest improvements.	Improving the Readability of Pediatric Hospital Medicine Discharge Instructions	. percentage of discharge instructions written at 7th grade or lower readability	multifaceted:1) education and implementation of a general discharge instruction template in the electronic health record (EHR); 2) visible reminders and tips for writing readable discharge instructions; 3) implementation of disease-specific discharge instruction templates in the EHR; and 4) individualized feedback to staff on readability and content of their written discharge instructions.	1a 2b	2a	64
Yin, H. S. 2017	USA	RCT	To determine whether parents who use a low literacy, pictogram- and photograph-based written asthma action plan have a better understanding of child asthma management compared to parents using a standard plan.	8,9	Use of a low literacy written asthma action plan was associated with better parent understanding of asthma management	Use of a low-literacy written action plan to improve parent understanding of pediatric asthma management: A randomized controlled study	. Errors in knowledge of medications	. pictogram- and photograph-based written asthma action plan	1a	1a	65
Hicks, B. L.2019	USA	quasi-experimental	To examine the impact of group-based central venous catheter education (CVC) on the knowledge, skill and comfort of caregivers, length of stay (LOS) related to initial CVC placement and 30-day return hospital visit for central line associated blood stream infections (CLABSI)	8,9	Group-based, education with use of the GLAD Model should to be considered as an effective educational intervention in providing caregivers CVC education	Group Style Central Venous Catheter Education Using the GLAD Model	.skill, knowledge and comfort<br>. length of stay<br>.30-day return hospital visit	.group-based education (GLAD Model) with enhanced educational materials and teachingmethods to alignwith adult learning practices	1ab	1ac	66

Khan, A.2018	USA	quasi-experimental	To determine whether medical errors, family experience, and communication processes improved after implementation of an intervention to standardize the structure of healthcare provider-family communication on family centered rounds	3,4,6,8,9	Although overall errors were unchanged, harmful medical errors decreased and family experience and communication processes improved after implementation of a structured communication intervention for family centered rounds coproduced by families, nurses, and physicians	Patient safety after implementation of a coproduced family centered communication programme: multicenter before and after intervention study	. Medical errors<br>. family satisfaction	. Families, nurses, and physicians coproduced an intervention to standardize healthcare provider-family communication on ward rounds: high reliability communication, written real-time summaries of rounds; a formal training programme for healthcare providers; and strategies to support teamwork, implementation, and process improvement	1ab2ab	1b2a	67
Lee, Jonathan S.2018	USA	quasi-experimental	To assess effects of a bedside interpreter-phone intervention on hospital discharge preparedness among patients with limited English proficiency (LEP).	7,9	Implementation of a bedside interpreter-phone systems intervention did not consistently improve patient-reported measures of discharge preparedness, possibly due to limited uptake during discharges	Hospital discharge preparedness for patients with limited English proficiency: A mixed methods study of bedside interpreter-phones	. discharge preparedness<br>. patient-reported knowledge<br>. Staff using interpreter-phones	a bedside interpreter-phone	1ab2b	1a2a	68
McEvoy, A.2018	Canada	Validation/usability	To produce and validate a set of illustrations for key counselling points of a pediatric eczema action plans (EAP)	2,8	A set of illustrations for use in an EAP was prospectively designed and validated, achieving acceptable transparency, translucency, and recall, with input from patients and a multidisciplinary medical team	Derivation, Evaluation, and Validation of Illustrations of Key Counselling Points for a Pediatric Eczema Action Plan	. transparency, translucency, and recall	. Developing illustration following the Children's Hospital of Eastern Ontario (Ottawa, ON, Canada) Asthma Action Plan and Anaphylaxis Action Plan protocols for validating illustrations/pictograms	1a	1a3c	69
Merchant, R. C.2018	USA	RCT	To investigate how well or for how long knowledge is retained after a videos and pictorial brochures intervention	8	Study findings will guide ED-based delivery of HIV/AIDS and HIV testing information; that is, whether delivery modes (video or pictorial brochure) should be selected for patients by language and/or health literacy level	Facilitating HIV/AIDS and HIV testing literacy for emergency department patients: a randomized, controlled, trial	. knowledge and retaining this knowledge over 12 months	videos and pictorial brochures	1a	1a	70
Plate, J. F.2019	UK	quasi-experimental	To determine which patients utilize a web portal (MyChart), and to determine the impact of secure messaging on emergency department (ED) visits and readmissions within 90 days postoperatively.	7	MyChart use did not decrease the 90-day rate of return to the emergency department (ED) or readmissions	Utilization of an Electronic Patient Portal Following Total Joint Arthroplasty Does Not Decrease Readmissions	. 90-day ED return or readmission	an electronic messaging portal (MyChart) to allow patients to directly communicate with their healthcare provider	1a	1c	71

Scalia, P.2019	USA	Validation/usability	To user-test a web-based, interactive Option Grid decision aid ‘prostate-specific antigen (PSA) test: yes or no?’ to determine its usability, acceptability and feasibility with men of high and low health literacy	2,7,8	Men in our sample with limited health literacy had difficulty navigating the Option Grid, thus suggesting that the tool was not appropriately designed to be usable by all audiences.	User-testing an interactive option grid decision aid for prostate cancer screening: lessons to improve usability	. Understandability, acceptability	. An interactive, web-based Option Grid patient decision aid for considering whether or not to have a PSA test	1a	3c	72
Staynova, R. A.2019	Bulgaria	Validation/usability	To adapt and elaborate printed educational materials on GDM and to evaluate the usefulness of the produced training materials and pregnant women’s satisfaction	2,8	The provision of educational materials on GDM can enhance pregnant women’s health literacy as well as their responsibility, motivation and attitude to their personal health	Written Health Education Materials for Women with Gestational Diabetes Mellitus - Evaluation of Usefulness and Patients' Satisfaction	. Usefulness<br>. Patient satisfaction	. Developing an educational manual	1a	1b	73
Wallace, A. S.2019	USA	Descriptive	To evaluate an interactive tool designed to help patients communicate their social resources supportive of home recovery to health care providers	8,9	The D-CEGRM may be an efficient tool for patients to communicate access to social resources, and an effective facilitator of transitional care planning.	Social resource assessment: Application of a novel communication tool during hospital discharge	. patients as likely to have “inadequate” or “adequate” supportive resources for home self-management	. tool to facilitate patient communication of personal supportive resources with healthcare teams, the Colored Eco-Genetic Relationship Map (CEGRM)	1a	3a	74
Chan, B. 2015	USA	RCT	To test using hcahps an intervention in which participants received inpatient visits by a language-concordant study nurse and post-discharge phone calls from a language-concordant nurse practitioner to reinforce the care plan and to address acute complaints.	7,9	An inpatient standalone transition-ofcare intervention did not improve patient discharge experience	The Effect of a Care Transition Intervention on the Patient Experience of Older Multi-Lingual Adults in the Safety Net: Results of a Randomized Controlled Trial	. hcahps score<br>. CTM3 scores	. participants received inpatient visits by a language-concordant study nurse and post-discharge phone calls from a language-concordant nurse practitioner to reinforce the care plan and to address acute complaints	1b	1b3a	84

Cochrane, Belinda 2016	Australia	quasi-experimental	To explore the views of stakeholders, including patients and the healthcare team, on the feasibility, acceptability and barriers to a collaborative, multidisciplinary team-based care intervention ('TEAMcare')	9	The study protocol was abandoned prematurely due to clear lack of feasibility. Of 12 participants, 4 withdrew and none completed pulmonary rehabilitation (PR). The main reasons for non-participation or study withdrawal related to reluctance to attend PR (6 of 16) and the burden of increased appointments (4 of 16).	Implementation challenges in delivering team-based care ('TEAMcare') for patients with chronic obstructive pulmonary disease in a public hospital setting: a mixed methods approach	. qualitative feedback about the intervention in terms of feasibility, acceptability and barriers via structured and semi-structured interviews	. individualised COPD management plans were based on the COPD care algorithm and typically comprised assessment of patient needs, establishment of management goals, disease-specific education and implementation of self-management strategies via coordination of treatment services by the allied health staff on the treating team, proactive monitoring and review	1b	1b	85
Daley, C. M. 2010	Albany	quasi-experimental	To test a Transitional Care Program	7,9	Continual identification of system or process and communication gaps postdischarge helped improve the continuum of care. Key findings from this study include a 30-day readmission rate for the study group of 15%, with an expected rate of 20%. Observed mortality rate was 2% for the study group with an expected rate of 7%.	A hybrid transitional care program	.readmission whithin 39 days .mortality	Transitional Care Program: multifaceted (multidisciplinary team, patient education, patient HL screened, follow-up telephone call, Reconciliation of medications upon hospital admission, discharge, and during the 6-month follow-up)	1b	1c	86
Jack, B. W. 2009	USA	RCT	To test the effects of an intervention designed to minimize hospital utilization after discharge.	7,9	Primary outcomes were emergency department visits and hospitalizations within 30 days of discharge. Secondary outcomes were self-reported preparedness for discharge and frequency of primary care providers' follow-up within 30 days of discharge Participants in the intervention group (n = 370) had a lower rate of hospital utilization than those receiving usual care (n = 368)	A Reengineered Hospital Discharge Program to Decrease Rehospitalization A Randomized Trial	. emergency department visits and hospitalizations within 30 days of discharge . self-reported preparedness for discharge and frequency of primary care providers' follow-up within 30 days of discharge	. Multifaceted:A nurse discharge advocate worked with patients during their hospital stay to arrange follow-up appointments, confirm medication reconciliation, and conduct patient education with an individualized instruction booklet that was sent to their primary care provider. A clinical pharmacist called patients 2 to 4 days after discharge to reinforce the discharge plan and review medications	1b	1ac	87

Johnson, R. W. 2012	USA	quasi-experimental	To describe and explore implementation of the Patient Self-Determination Act in a critical care setting. The goal was to assess patients' understanding of advance directives	2,9	Of the patients who initially declined the opportunity to complete an advance directive (n = 213), 33.8% (n = 72) said they did not understand the question when initially asked and therefore just said no.	Reasons for noncompletion of advance directives in a cardiac intensive care unit	.understanding of advance directives	. implementation of the Patient Self-Determination Act asking "Do you have an advance directive?"	1b	1a	88
Masterson Creber, Ruth 2016	USA	RCT	To test the efficacy of a tailored motivational interviewing (MI) intervention versus usual care for improving Heart failure (HF) self-care behaviors, physical HF symptoms and quality of life	6, 7	Patients who received the MI intervention had significant and clinically meaningful improvements in HF self-care maintenance over 90 days that exceeded that of usual care	Motivational interviewing to improve self-care for patients with chronic heart failure: MITI-HF randomized controlled trial	.self-care maintenance over 90 days	. a tailored motivational interviewing (MI) intervention	1b	1c	89
Micklethwaite, A. 2012	USA	quasi-experimental	The 3-component program sought to improve meaningful access to care, increase health literacy related to type 2 diabetes, and partner with the enrollees to make long-term lifestyle changes. Intervention strategies included (1) diabetes education, (2) self-management skills training, and (3) diabetes case management services.	6, 7, 8, 9	The intervention not only resulted in significant improvements in HbA1c levels (-0.77%) but saved the hospital an average of $551 per active patient per year, primarily by reducing hospital visits.	The business case for a diabetes self-management intervention in a community general hospital	. improvements in HbA1c levels<br>. n. of hospital visits	. (1) diabetes education, (2) self-management skills training, and (3) diabetes case management services.	1b	1c	90
Oancea, C. 2015	Romania	quasi-experimental	The main objective of this study was to assess the effects of intensive medical education courses on chronic obstructive pulmonary disease (COPD)related rehospitalizations and emergency department visits and the secondary aim was to monitor quality of life.	9	This study reveals that an adequate medical education program is associated with a decreased rate of COPD-related hospitalizations. The implementation of a medical education program as an integrated part of therapy could lead to a more accurate self-management of the disease.	Impact of medical education program on COPD patients: a cohort prospective study	. rate of COPD-related hospitalizations	. intensive medical education courses	1b	1c	91

Stockwell, M. S. 2014	USA	quasi-experimental	To evaluate the effectiveness of an educational intervention to decrease pediatric emergency department (PED) visits and adverse care practices for upper respiratory infections (URI) among predominantly Latino Early Head Start (EHS) families	9	A URI health literacy-related educational intervention embedded into EHS decreased PED visits and adverse care practices.	Effect of a URI-related educational intervention in early head start on ED visits	.decreased PED visits and adverse care practices	.educational intervention(three 1.5-hour education modules)	1b	1c	92
Wolf, M. S. 2014	USA	RCT	To compare two implementation approaches for a health literacy diabetes intervention designed for community health centers	6,8,9	An outsourced diabetes education and counseling approach for community health centers appears more feasible than clinic-based models. Patients receiving the carve-out strategy also demonstrated better clinical outcomes compared to those receiving the carve-in approach.	Clinic-based versus outsourced implementation of a diabetes health literacy intervention	. Diabetes knowledge<br>. Self-efficacy<br>. Health behaviors<br>. HbA1C, mean<br>. Systolic blood pressure, mean<br>. Diastolic blood pressure, mean<br>. LDL cholesterol, mean	. education and counseling	1b	1ac	93
Davis, J. 2017	USA	case study	To implement a standardized patient engagement strategy to assess health literacy using Hospital Consumer Assessment of Healthcare Providers and Systems (HCAHPS) scores to measure improvement in the domain of RN Communication	2,3,6	The HCAHPS scores improved during the trial period, which indicated that implementing this protocol had positive implications for patient satisfaction.	Engage(2): Implementing a Health Literacy Protocol for Patient Assessment and Engagement	. HCAHPS scores	.patient engagement strategy (two verbal questions, developed specifically for the hospital setting, that would bypass written assessment for health literacy and initiate positive interactions between nurses and patients.	1b	1b3a	94
Dingemans, A. J. M. 2017	USA	quasi-experimental	To investigate whether a timed health literacy intervention could improve health literacy in this population	9	Emphasis on providing an education-based approach at each visit increased health literacy significantly. As expected, health literacy was lowest during the first visit, which we believe is the optimal time to implement educational interventions.	Does clinic visit education within a multidisciplinary center improve health literacy scores in caregivers of children with complex colorectal conditions?	. Health literacy level	. education-based approach at each visit	1b	1a	95
Lee, J. S. 2017	USA	quasi-experimental	To assess the impact of a bedside interpreter phone system intervention on informed consent for patients with LEP and compare outcomes to those of English speakers)	6,9	Bedside interpreter phone system intervention to increase rapid access to professional interpreters was associated with improvements in patient-reported informed consent	Increased Access to Professional Interpreters in the Hospital Improves Informed Consent for Patients with Limited English Proficiency	. adequately informed consent	. bedside interpreter-phone	1b	1a	96

Navaneethan, S. D. 2017	USA	RCT	To develop a navigator program for patients with CKD and an electronic health record–based enhanced personal health record to disseminate CKD stage–specific goals of careand education	7	There were no differences in eGFR decline and other outcomes among the studygroups	Pragmatic randomized, controlled trial of patient navigators and enhanced personal health records in CKD Pragmatic randomized, controlled trial of patient navigators and enhanced personal health records in CKD	. change in eGFR over a 2-year follow-up period<br>. acquisition of appropriate CKD-related laboratory measures<br>. specialty referrals<br>. hospitalization rates	. a navigator program for patients with CKD and an electronic health record–based	1b	1c	97
Kripalani, S.2019	USA	quasi-experimental	To evaluate two forms of an evidence-based, multi-component transitional care intervention	7,9	An evidence-based multi-component intervention delivered by nurse TCCs reduced 30- and 90-day<br>readmissions and associated health care costs.	A transition care coordinator model reduces hospital readmissions and costs	. 30- and 90-day readmissions<br>. health care costs	. Transition Care Coordinator (TCC)	1b	1c3d	98
Rayan-Gharra, N.2019	Israel	quasi-experimental	To examine whether hospital discharge practices and care-transition preparedness mediate the association between patients' cultural factors and readmissions	6,7,9	The results show that the association between patients' cultural factors and 30-day readmission is mediated by the hospital discharge practices and care transition	Association between cultural factors and readmissions: the mediating effect of hospital discharge practices and care-transition preparedness	. 30-day readmission	. discharge planning tailored to patients' cultural characteristics	1b	1c	99
Grice, G. R. 2014	USA	quasi-experimental	Evaluate curricular changes related to health literacy and determine impact on independent-living senior residents as part of an introductory pharmacy practice experience for third-year student pharmacists	3,6	Outcomes included resident satisfaction, student satisfaction, and correlations between student use of health literacy tools and resident satisfaction	Student use of health literacy tools to improve patient understanding and medication adherence	.resident satisfaction<br>.student satisfaction<br>.correlations between student use of health literacy tools and resident satisfaction	. three methods: Ask Me 3™, Four Habits Model, and Teach-back (Interpersonal Communications)	2a	2b	100
Kornburger, C. 2013	USA	quasi-experimental	To evaluate an educational intervention for nurses on "teach-back" which encouraged nurses to check for patients' and caregivers' understanding of discharge instructions prior to discharge.	3,6	Pre and post survey data collected from nurses specifically demonstrated the positive effect "teach-back" could have on preventing medication errors while also simultaneously identifying areas for further study	Using "Teach-Back" to Promote a Safe Transition From Hospital to Home: An Evidence-Based Approach to Improving the Discharge Process	.increased knowledge regarding health literacy and "teach-back"<br>.change in nurse behavior and practice<br>.usefulness of teach-back (nurses)	. ducational intervention for nurses on "teach-back"	2a	2ab	101

Krajic, K. 2005	USA	quasi-experimental	Cultural competence training was provided for all types of hospital staff, primarily with the intention of providing support for staff. The evaluation criteria were feasibility/acceptability, quality, effectiveness, cost-effectiveness and sustainability	3,6	Key findings showed that seven of the eight pilot hospitals managed to implement cultural competence training. Acceptance of the training among staff, measured in terms of participation, varied considerably. Variations were also identified in the quality of the training as measured by concordance with an agreed implementatio pathway. The training had a positive impact on staff perceptions of their knowledge, skills and comfort levels in transcultural situations. The training was also considered to be cost-effective with regard to external costs, and sustainable in that it was accepted as part of continuous professional development in hospitals. the training among staff, measured in terms of participation, varied considerably.Variationswere also identified in the quality of the training as measured	Improving ethnocultural competence of hospital staff by training: experiences from the European 'Migrant-friendly Hospitals' project	.acceptance of the training among the staff<br>.Perception of of their knowledge, skills and comfort levels in transcultural situations<br>.training partecipation<br>.quality of training	.cultural competence training for staff	2a	2ab	102
Kripalani, S. 2006	USA	Validation/usability	To evaluate a training program for medical residents that includes a videotaped standardized patient encounter, interactive small-group workshop, one-on-one feedback with a faculty member, and an individual behavioral prescription for improved communication	3	Residents felt that the topic was relevant and that their communication skills benefited from the intervention. They enjoyed the teaching methods, particularly the individual feedback on their videotaped encounter	Development and Implementation of a Health Literacy Training Program for Medical Residents	.perception of increased communication skills<br>.enjoyed the teaching methods	. a training program for medical residents	2a	2ab	103
Lori, Jody R. ; 2016	Ghana	Descriptive	The purpose of this pilot study is to examine the usefulness and feasibility of providing focused antenatal care (FANC) in a group setting using a manualized intervention to improve patient-provider communication, patient engagement, and improve health literacy.	3,6	Quantitative survey results indicate that midwives believe they are doing a good job communicating with and engaging antenatal patients regardless of whether care is provided individually or in a group format. However, focus group data provide a more comprehensive assessment of maternal health literacy and antenatal care.	Use of a facilitated discussion model for antenatal care to improve communication	. perceived patient-provider communication and engagement	. A training of trainers (TOT) was conducted with the six midwives. Each midwife administered seven modules to two groups over the course of establishing fidelity for a total of 14 group visits.	2a	2b	104

Shipman, J. P. 2009	USA	quasi-experimental	To evaluate the health literacy curriculum and its impact on the target group	1,3	Participation in a curriculum increased health care providers' knowledge of health information literacy, awareness of available consumer health information, and referral of patients to the library for additional assistance	The health information literacy research project	. awareness of available consumer health information<br>. knowledge of health information literacy<br>. Patient referred to of MedlinePlus or hospital librarian	. health literacy curriculum	2a	2a	105
Stikes, R. 2015	USA	descriptive study	To describe how participation in the Sigma Theta Tau International Maternal-Child Health Nurse Leadership Academy positioned the authors to lead an interdisciplinary team through implementation and evaluation of a change project related to patient education based upon national health literacy standards. The project goal was to improve patient satisfaction with nurse communication and preparation for hospital discharge	1,2,3	The leadership academy successfully positioned the authors to guide an interdisciplinary team through development of a process to meet the education and communication needs of patients and improve their health literacy. As a result, a positive effect was noted on patient satisfaction with health communication	A Nurse Leadership Project to Improve Health Literacy on a Maternal-Infant Unit	.patient satisfaction whith health communication	. participation in the Sigma Theta Tau International Maternal-Child Health Nurse Leadership Academy	2a	1b	106
Trummer, U. F. 2006	Austria	Descriptive	To evaluated were the effects of a training program for developing communication skills of health professionals (physicians, physiotherapists, and nurses) along with a reorganization of patient information schemes.	2,3,6	In the intervention group length of hospital stay was shorter (by 1 day), incidence of post-surgery tachyarrhythmia was reduced (by 15%), transfer to less intensive care levels was faster and patient ratings for communicative quality of care by doctors and nurses were improved.	Does physician-patient communication that aims at empowering patients improve clinical outcome? A case study	.level adjusted length of stay in hospital<br>. frequency of post-surgery complication<br>. subjective health<br>.subjective satisfaction with care received	.multifaceted: training program for developing communication skills of health professionals along with a reorganization of patient information schemes	2a	1bc	107
Grabeel, K. L.2018	USA	Descriptive	To assess the health literacy knowledge of hospital clinical staff. Additionally, researchers wanted to promote the importance of health literacy to hospital personnel	1,2,3	The result is an increased dialogue of health literacy at the hospital, along with new research projects, a forum, and a summit meeting.	Taking the Pulse of the University of Tennessee Medical Center's Health Literacy Knowledge	. Staff requesting health literacy training<br>. Staff trainings<br>. Team meetings	. librarians conducted a needs assessment of hospital staff to determine their knowledge of health literacy and the need for training.<br>. As a follow-up, library staff conducted training sessions for nurses through classes, small group meetings, and staff huddles.	2a3a	2a3b	108

Klingbeil, C.2018	USA	Descriptive	To examine the impact of a brief educational intervention for a multidisciplinary staff on knowledge of health literacy and the use of teach-back during patienteducation	3,6	Teach-back is a valuable strategy that can improve the safety and quality of health care and supports the National Action Plan to Improve Health Literacy.	The Teach Back Project: A System-wide Evidence Based Practice Implementation	. knowledge of the teach-back process<br>. rates of clarifying information	. brief educational intervention for a multidisciplinary staff on knowledge of health literacy and the use of teach-back	2a	2a	109
Roberts, N. J. 2010	UK	quasi-experimental	Usage of a pictorial software for help asthma patients was assessed and health care professionals questioned as to its use.	2,3,8	It is to be hoped that such facilitation enhances the number of action plans issued and in this study GPs were greater users than the nurses	Development of an electronic pictorial asthma action plan and its use in primary care	. usage: n. of plans printed	. an electronic pictorial asthma action plan (E-PAAP)	2b	2a	110
Beauchamp, Alison 2017	Australia	Descriptive	To test the Ophelia approach	1,2	Changes were observed at organisational, staff, and community member levels. The Ophelia approach can be used to generate health service improvements that enhance health outcomes and address inequity of access to healthcare	Systematic development and implementation of interventions to OPtimise Health Literacy and Access (Ophelia)	. Interventions generated<br>. HLQ scales<br>. interviews with clients or staff<br>. Staff's focus groups	. Ophelia approach: each site identified their own priorities for improvement; collected health literacy data using the Health Literacy Questionnaire (HLQ) within the identified priority groups; engaged staff in co-design workshops to generate ideas for improvement; developed program-logic models; and implemented their projects using Plan-Do-Study-Act (PDSA) cycles	3ab	3ab	111
Gazmararian, J. A. 2010	USA	Validation/usability	To develop a health literacy organizational assessment tool for health plans that could be used as a benchmark and encourage further work	2,7,8	Feedback from the participating plans has been very positive about using this tool as a benchmark and using the results to help strengthen health literacy efforts within their health plans.	The Development of a Health Literacy Assessment Tool for Health Plans	.A summary of some of the overall responses for each section of the assessment tool	. Use of the developed health literacy organizational assessment tool	3a	3a	112
Innis, Jennifer 2017	Canada	Validation/usability	To measure health literate discharge practices in Ontario hospitals using a new organizational survey questionnaire tool and to perform psychometric testing of this new survey	2,9	The survey could be used by managers to measure health literate discharge practices in hospitals to obtain a baseline measure, which identifies areas that need improvement and that could be addressed through quality improvement plans. Survey results could also be used to develop staff education sessions	Measuring health literate discharge practices	. health literate score<br>. Survey reliability	. organizational survey questionnaire tool	3a	3a	113

Kowalski, C. 2015	Germany	Validation/usability	To present the effort in developing and validating an HLHO instrument.	2,4	The instrument has satisfactory reliability and validity. It provides a useful tool to assess the degree to which health care organizations help patients to navigate, understand, and use information and services	The health literate health care organization 10 item questionnaire (HLHO-10): development and validation	.instrument reliability and validity	. development and validation of the health literate health care organization 10 item questionnaire (HLHO-10)	3a	3a	11
Weidmer, B. A. 2012	USA	Validation/usability	To develop a set of items to supplement the CAHPS® Hospital Survey (HCAHPS) to assess how well hospitals communicate health information to inpatients.	1,2	This study provides support for the measurement properties of the HCAHPS Item Set for Addressing Health Literacy	Development of items to assess patients' health literacy experiences at hospitals for the Consumer Assessment of Healthcare Providers and Systems (CAHPS) Hospital Survey	. Correlations between items and scales with hypothesized item cluster	.development a set of items to supplement the CAHPS® Hospital Survey (HCAHPS)	3a	3c	114
Oelschlegel, Sandy 2018	USA	Descriptive	The article discusses one medical library's process of leading systematic assessment of their organization's health literacy attributes	1,2	This project may serve as a model for other medical librarians.	Librarians Promoting Changes in the Health Care Delivery System through Systematic Assessment	. Health Literacy Environment of Hospitals and Health Centers (HLEHHC)score	. library's process of leading systematic assessment of their organization's health literacy attributes	3a	3a	115
Palumbo, R. 2017	Italy	Descriptive	To delve into the meaningfulness of health care organizations, embracing the "organizational health literacy" perspective	1,2	The health care organizations were unaware of several crucial issues to improve their meaningfulness. Problematic organizational health literacy was found to prevent patient involvement and tonegatively affect the quality of interaction between the patients and the health care professionals	Exploring the meaningfulness of healthcare organizations: a multiple case study	. Health Literacy Environment of Hospitals and Health Centers (HLEHHC)score<br>. Documents about the role of organizational health literacy in the policies and strategies of health care organization<br>. interviews were realized to collect first-hand evidence of the factual implementation of organizational health literacy initiatives	. document analysis concerned strategic and business plans, policy reports, information pamphlets, and internal documents<br>. unstructured interviews<br>. an assessment of the organizational health literacy (Health Literacy Environment of Hospitals and Health Centers (HLEHHC)score)	3ab	3ab	116
Hayran, O.2018	Turkey	quasi-experimental	To assess the organizational health literacy (OHL) of a group of hospitals and investigate the relationships among OHL, patient satisfaction, and patients' health literacy	2	OHL seems to be a significant determinant of patient satisfaction	Organizational health literacy as a determinant of patient satisfaction	. patient satisfaction<br>. HLHO-10 questionnaire consistency	. filling out the 'Health Literate Health care Organizations-10' (HLHO-10)	3a	1b3ac	117

Jessup, R. L.2018	Australia	quasi-experimental	The aim of this study is to describe a staff and patient co-design process that will lead to the development of health literacy interventions in response to identified health literacy needs of hospital patients	4,8	This study identified fifteen strategies to address health literacy needs of a hospital population. Implementation and evaluation will identify sets of strategies that have the maximum patient, clinician and organisational benefit.	Using co-design to develop interventions to address health literacy needs in a hospitalised population	. key themes identified<br>. interventions produced	. Step 1: hospitalised patients surveyed and data analysed using hierarchical cluster analysis to establish health literacy profiles<br>. Step 2: clusters presented as vignettes to patients and clinicians to co-design interventions to address needs.	3ab	3b	118
Innis, J. 2017	USA, Canada	Descriptive	A Delphi panel consisting of nurses, other health care providers, and researchers was used to develop a set of indicators of health literate discharge practices based on the practices of Project RED (Re-Engineered Discharge).	2,9	This study led to the development of indicators that can be used to evaluate the use of HLDPs.	Development of Indicators to Measure Health Literate Discharge Practices	.developed indicators for discharge practice	. A Delphi panel consisting of nurses, other health care providers, and researchers was used to develop a set of indicators of health literate discharge practices based on the practices of Project RED (Re-Engineered Discharge).	3b	3b	119
Seligman, Hilary K. 2007	USA	Descriptive	To develope a theoretically informed process for developing educational materials	8	Developing low-literacy health education materials that will activate patients toward healthier behaviors requires attention to factors beyond reading level.	Facilitating behavior change with low-literacy patient education materials	. Satisfaction with the new guide	. informed process for developing educational materials	3b	1b	120
Chin, J. 2017	USA	Validation/usability	To develop a systematic approach to improve web-based health information so that it is better understood and used by older adults with varying health literacy abilities.	2,7,8	A systematic, multi-faceted approach to designing health documents can promote online learning among older adults with diverse health literacy abilities.	A Multi-faceted Approach to Promote Comprehension of Online Health Information Among Older Adults	. reduce comprehension demands on health-literacy-related cognitive resources<br>. Reading Efficiency (question accuracy, summary accuracy)	.redesigning health documents	3b	1a3a	121

Naccarella, Lucio2019	Australia	Descriptive	To provides insights into a practice-based initiative by Heart Foundation Victoria to improve access to recovery information for patients with low literacy levels	2,7,8	Strategies to improve recovery through increased heart health literacy must address the needs of patients, nursing staff and the health system within hospitals	Improving access to important recovery information for heart patients with low health literacy: reflections on practice-based initiatives	. increased access to recovery information<br>. nurse knowledge and confidence to engage with patients on recovery information<br>. improved education of patients and improved availability and accessibility of information for patients in diverse formats	. development of recovery information for patients with low literacy<br>. development of a standard resource and associated protocols for cardiac nurses<br>.development of tailored resources, training and networking opportunities, as well as improving hospital protocols and documentation.	3b	1a2a3b	122

Table S2: Systematic reviews

**Author, year**	**Aim**	**N. studies**	**Attributes**	**Conclusions**	**Title**	**Intervention**	**Outcome**	**Intervention code**	**Outcome code**	**Ref**

Armstrong, B. 2011	To examine: (a) the interventions utilized to provide discharge instructions, (b) the methods used to assess patient comprehension, and (c) the most effective strategies for assuring patient comprehension of ED discharge instructions	**21**	8,9	Increases in patient comprehension of ED discharge instructions are identified with alternative teaching interventions (multimedia) in comparison to traditional standardized written discharge instructions (usual care). Literature suggests clinicians seldom clarify patient comprehension in practice, though effective methods are noted in research	Patient comprehension of discharge instructions from the emergency department: A literature review	Discharge teaching interventions: standard or usual care ; handwritten discharge materials;oral instructions combined with written directions; diagnosis specific; or pamphlet use of discharge instructions, Multimedia teaching interventions; use of a bilingual discharge facilitator	. patient comprehension:	1ab	1a	75
Berkman, N. D. 2011	To review interventions designed to improve these outcomes for individuals with low health literacy. Disparities in health outcomes and effectiveness of interventions among different sociodemographic groups were also examined	42	8,9	Among intervention studies (27 randomized controlled trials [RCTs], 2 cluster RCTs, and 13 quasi-experimental designs), the strength of evidence for specific design features was low or insufficient. However, several specific features seemed to improve comprehension in one or a few studies. The strength of evidence was moderate for the effect of mixed interventions on health care service use; the effect of intensive self-management inventions on behavior; and the effect of disease-management interventions on disease prevalence/severity. The effects of other mixed interventions on other health outcomes, including knowledge, self-efficacy, adherence, and quality of life, and costs were mixed; thus, the strength of evidence was insufficient.	Health literacy interventions and outcomes: an updated systematic review	. Alternative material design<br>. Physician Notification of Patient Literacy Status<br>. Patient education	. Patient knowledge<br>. Self-Efficacy<br>. Patient skill<br>. Patient Behaviors<br>. Quality of life<br>. Medication Adherence<br>. Disease prevalence and severity	1a2b	1ac	76

Chan, H. K. 2015	to determine the effectiveness of pictorial aids used to assist caregivers in the administration of liquid medications	5	8	The existing findings suggest that pictographic interventions reduced dosing errors, enhanced comprehension and recall of medication instructions and improved adherence of caregivers.	Using pictograms to assist caregivers in liquid medication administration: a systematic review	. Pictorial aids for patients/caregivers	. Dosing errors<br>. Patient comprehension<br>. Recall of medication instructions<br>. Improved adherence of caregivers	1a	1a	77
Jacobs, R. J. 2014	to review empirically based eHealth intervention strategies designed to improve health literacy among consumers in a variety of settings	12	6,8	Compared to control interventions, the interventions using technology reported significant outcomes or showed promise for future positive outcomes regarding health literacy in a variety of settings, for different diseases, and with diverse samples	A systematic review of eHealth interventions to improve health literacy	. eHealth platforms with web-based applications that included multimedia applications such as videos and interactive self-help tools	. Patient knowledge<br>. Patient behaviors<br>. Perceived risks<br>. Disordered eating<br>. Excessive exercise<br>. Body dissatisfaction	1a	1a	78
Santo, Anelise 2005	To evaluate the efficacy of audiotapes as a health information exchange intervention, specifically looking for use with the ‘‘hardto- reach’’ population	35	6,8	Audiotapes were used to record consultations and health-related information in specific health situations. No studies were found that targeted the needs of the ‘‘hard-to-reach’’ population.With the exception of positive patient satisfaction, the benefits of audio taped messages remain unclear	Exploring the value of audiotapes for health literacy: A systematic review	. audiotapes as a health information	. knowledge and recall<br>. behavioral change<br>. Anxiety<br>. self-care<br>. satisfaction	1a	1ab	79

Sheridan, S. L. 2011	defining the current state of the literature about interventions designed to mitigate the effects of low health literacy	38	3,5,6,8,9	Multiple discrete design features that improved comprehension in one or a few studies (e.g., presenting essential information by itself or first, presenting information so that the higher number is better, adding icon arrays to numerical information, adding video to verbal narratives). In a few studies, we also found consistent, direct, fair or good-quality evidence that intensive self-management interventions reduced emergency department visits and hospitalizations; and intensive self- and disease-management interventions reduced disease severity. Evidence for the effects of interventions on other outcomes was either limited or mixed. Multiple interventions show promise for mitigating the effects of low health literacy and could be considered for use in clinical practice	Interventions for Individuals with Low Health Literacy: A Systematic Review	. Material design<br>. Multimedia<br>. Education/training for patient<br>. Helath literacy training for physicians	. Costs<br>. Quality of life<br>. Disease severity<br>. Medication adherence<br>. Behavior<br>. Skill<br>. Self efficacy<br>. Knowledge<br>. Hospitalizations<br>. Emergency department visits<br>. Use of services	1ab2a	1ac3d	80
Wilson, E. A. H. 2012	Evaluate the evidence regarding the relative effectiveness of multimedia and print as modes of dissemination for patient education materials; examine whether development of these materials addressed health literacy.	30	8	Multimedia appears to be a promising medium for patient education; however, the majority of studies found that print and multimedia performed equally well in practice. Few studies involved patients in material development, and less than half assessed the readability of materials	Comparative analysis of print and multimedia health materials: A review of the literature	. print and multimedia health materials	. Preference<br>. Knowledge	1a	1ab	81
Curran, J. A.2019	to examine how and why discharge communication works in a pediatric ED context and develop recommendations for practice, policy, and research	75	6,7,8,9	Strengthening discharge communication in a pediatric emergency context presents a significant opportunity for improving parent comprehension and health outcomes for children	Discharge communication practices in pediatric emergency care: a systematic review and narrative synthesis	. Patient education<br>. Patient incentives<br>. Environmental restructuring<br>. Modeling<br>. Enablement	. Knowledge<br>. Adherence<br>. Skill	1ab	1a	82

Tan, J. P.2019	To evaluate the effectiveness of educational interventions in improving medication adherence among adult patients diagnosed with hypertension, hyperlipidaemia and/or diabetes	18	8,9	Through education, health literacy is improved, thus improving medication adherence	A systematic review and meta-analysis on the effectiveness of education on medication adherence for patients with hypertension, hyperlipidaemia and diabetes	. Educational face-to-face<br>. Educational material	. Medication adherence	1ab	1a	83
